# Using institutional theory to analyse hospital responses to external demands for finance and quality in five European countries

**DOI:** 10.1177/1355819615622655

**Published:** 2015-12-17

**Authors:** Susan Burnett, Peter Mendel, Francisco Nunes, Siri Wiig, Hester van den Bovenkamp, Anette Karltun, Glenn Robert, Janet Anderson, Charles Vincent, Naomi Fulop

**Affiliations:** 1 Researcher, Department of Surgery, Faculty of Medicine, Centre for Patient Safety and Service Quality, Imperial College London, UK; 2 Senior Sociologist, Rand Corporation, Santa Monica, CA, USA; 3 Assistant Professor of Human Resources and Organizational Behaviour, ISCTE, Lisbon University Institute, Portugal; 4 Professor of Quality and Safety in Healthcare Systems, Department of Health Studies, University of Stavanger, Norway; 5 Associate Professor of Public Administration in Health Care, Institute of Health Policy & Management, Erasmus University Rotterdam, The Netherlands; 6 Assistant Professor, The Jönköping Academy for Improvement of Health and Welfare, Sweden; 7 Professor of Healthcare Quality & Innovation, Florence Nightingale Faculty of Nursing and Midwifery, King’s College London, UK; 8 Senior Lecturer, Florence Nightingale Faculty of Nursing and Midwifery, King’s College London, UK; 9 Professor of Psychology, Department of Experimental Psychology, Oxford University, UK; 10 Professor of Health Care Organisation and Management, Department of Applied Health Research, University College London, UK

**Keywords:** finance, health care, institutional theory, quality

## Abstract

**Objectives:**

Given the impact of the global economic crisis, delivering better health care with limited finance grows more challenging. Through the lens of institutional theory, this paper explores pressures experienced by hospital leaders to improve quality and constrain spending, focusing on how they respond to these often competing demands.

**Methods:**

An in-depth, multilevel analysis of health care quality policies and practices in five European countries including longitudinal case studies in a purposive sample of ten hospitals.

**Results:**

How hospitals responded to the financial and quality challenges was dependent upon three factors: the coherence of demands from external institutions; managerial competence to align external demands with an overall quality improvement strategy, and managerial stability. Hospital leaders used diverse strategies and practices to manage conflicting external pressures.

**Conclusions:**

The development of hospital leaders’ skills in translating external requirements into implementation plans with internal support is a complex, but crucial, task, if quality is to remain a priority during times of austerity. Increasing quality improvement skills within a hospital, developing a culture where quality improvement becomes embedded and linking cost reduction measures to improving care are all required.

## Introduction

Delivering better health care with limited finance is arguably more of a challenge today than ever before in Europe^1^ and other developed countries^2^ given the impact of the global economic crisis and our increasing knowledge about how to improve the quality of care. We report the findings from the Quality and Safety in European Hospitals (QUASER) study, an EU-funded multilevel study of 10 hospitals in five European countries (England, the Netherlands, Norway, Portugal and Sweden). The study was designed to investigate how hospitals working in different systems implement, spread and sustain quality improvement (QI), including the difficulties they face and how they overcome them. This paper applies a framework that draws on institutional theories^3,4^ to explore how hospital leaders balance external pressures to improve quality and constrain spending.

QI has been defined as ‘better patient experience and outcomes, achieved through changing provider behaviour and organization, through using systematic change methods and strategies’.^5^ Most contemporary QI approaches in health care have their roots in the 1940s and 1950s, informed by experts such as Juran and Deming who were considering ways to manage the quality of manufacturing production.^6^ Beginning in the US in the early 1990s, the application of QI in the health care sector has become more systematic.^7^ However, until recently, one criticism of QI in health care remained: that it was ‘under-theorized and over-popularized’.^8^ Drawing particularly on perspectives from the social sciences, greater attention has begun to be paid to the processes of implementing and sustaining QI efforts. This has led to the present-day conceptualization of this, now global, field as ‘Improvement Science’, where a more scientific approach to improvement is proposed as having the potential to ensure both high-quality and efficient care.^9^ Drawing on this, the current study used the ‘Organizing for Quality’ framework^8^ as the dimensions underpinning the work done by hospital leaders to develop and embed QI activity, as follows:

- Structural – structuring, planning and co-ordinating quality efforts
- Political – addressing the politics of change, negotiating buy-in, resolving conflict surrounding any QI effort
- Cultural – giving ‘quality’ a shared, collective meaning, value and significance
- Educational – creating and nurturing a learning process
- Emotional – inspiring, energizing and mobilizing people for QI
- Physical and technological – designing systems and infrastructures that support QI
- Leadership – providing clear, strategic direction
- External demands – responding to and managing the broader social, political and contextual factors

These dimensions show that QI does not happen in a vacuum and that a range of internal and external influences need to be studied to understand how QI works in organizations. Institutional theory provides conceptual frameworks for examining the nature of external demands and the internal reactions of organizations.^3^ For example, in the seminal study of the US health care field, Scott^10^ examined how institutional pressures emanating from multiple entities may differ and compete (e.g. legislative, professional, accreditation, funders), thereby creating conflict and variance at the industry and organizational levels (e.g. individual hospital).

Institutional analysts have highlighted how organizational responses to external pressures and resource dependencies may vary across contexts, and how organizational leaders exercise a range of strategic choices.^4,11^ Kraatz and Block^12^ described four strategies from eliminating the source of external pressure to forging a new institutional order. Others^13,14^ have considered how responses to external demands are shaped by intra-organizational dynamics and the nature of external demands. A model developed by Oliver^4^ usefully identified a continuum of responses to understand how organizations respond to competing demands:

- Acquiescence: organizations comply with institutional demands whether through habit, imitation or conscious decision (for example, because the leaders agree with the demands).
- Compromise: organizations conform to the spirit, if not the letter of the demands, by either adjusting demands and/or internal responses. Compromise may arise by: balancing competing expectations via negotiating with internal groups; allocating energies to pacify those resisting or bargaining with external institutions.
- Avoidance strategies involve attempts by organizations to adjust conditions so as to make it possible for them to appear to comply with external demands. Tactics include: concealing non-conformity by symbolically or rhetorically ‘pretending’ to acquiesce; preventing technical monitoring of compliance (buffering) or by changing an organizational function so as to make compliance unnecessary (escaping).
- Defiance occurs when organizations reject external demands and may be manifested as dismissal of a demand or overtly challenging a requirement.
- Manipulation refers to the deliberate attempt actively to change the content of external demands. This may involve lobbying to control of the source of pressure or to generate demands that are beneficial (for example, to help improve quality of care).

Having examined the different models in relation to our data, the model by Oliver^4^ was selected as the most appropriate to develop our understanding of how hospital leaders respond to the competing external pressures of constraining spending while improving the quality of care. Through this analysis, we propose a typology of strategic responses by hospitals, taking into account their internal and external environments/characteristics. Using this typology, lessons are identified for senior hospital leaders and policy makers.

## Methods

Quality was defined as comprising clinical effectiveness, patient safety and patient experience and conceptualized as a human, social, organizational and technical accomplishment. Research teams from universities in each country participated using a common research protocol.^15^ If required, ethical approval was granted in each country. The countries were chosen to represent variation in important aspects of health care, such as funding arrangements and health care quality.^15^

The methods were designed for the overall QUASER study, described by Robert et al.^15^ This involved an in-depth, multilevel (national, organizational and clinical micro-system) analysis of health care quality policies and practices in each country, including longitudinal case studies in a purposive sample of 10 hospitals, two in each country. A case study was defined as an in-depth study of a relatively bounded phenomenon where the aim is to elucidate the features of a larger class of similar phenomena.^16^ Cross-case, comparative analysis, particularly across different contexts, is especially valuable in exploring similarities, commonalities and differences, thereby strengthening explanatory power.^17^

The hospital selection process was designed to find hospitals at different stages of QI, rather than only those seen to be performing well. A range of publicly available indicators of the process and outcome of care were used for the selection, together with information from the regulation/accreditation of hospitals. A full description of the selection process is described by Burnett et al.^18^

Data collection and analysis used a preliminary theoretical framework rather than a purely grounded theory approach^19^ so that data analysis was a combination of induction (data-driven) and deduction (theory-driven).^20^ Building on earlier findings from Bate et al.,^8^ the meso- and micro-system fieldwork, and the analysis of the wider health care system, sought an in-depth understanding of the processes that enable hospitals in Europe to achieve improvements in quality over time.

### Data collection

Data relating to the national context were collected from documentary sources using an agreed structure,^21^ covering the period of the research. This information included funding; access; the regulatory framework; accreditation; monitoring and information availability.

At the meso- and micro-system level in the 10 hospitals, the research teams conducted a total of 387 interviews (217 senior leaders; 170 frontline clinicians) and 796 h of observation of meetings and activities related to QI work from April 2011 to June 2012. The interview protocols were based on the ‘Organizing for Quality Framework’^8^ augmented by two additional ‘challenges’ – ‘leadership’ and ‘external demands’^15^ as set out in the introduction. Interview data on the first seven dimensions were used to characterize the hospitals’ strategic choices and tactics reflected in their QI programs. For external demands, we focused on responses from hospital leaders (clinicians and managers) to questions specifically relating to finance and quality:

- Has the current financial context impacted on the QI work undertaken in the hospital?
- Do senior leaders explicitly consider the financial implications of ‘doing’ or ‘not doing’ QI?
- To what extent do requirements of government, accrediting organizations or payers determine the selection and use of quality indicators?
- How much of what happens in QI is determined within the hospital in contrast to responding to external targets and priorities?
- Which national/regional policies support/hinder the hospitals pursuit of quality?

### Data analysis

Using the common framework, interviews and observation notes were coded by research teams in each country. First, each hospital (labelled A and B, below) was analysed separately, then they were compared.^17^ Hospitals were selected as being at different stages of performance with regard to quality, so the within-country pair-wise comparison looked at how they differed in their approach to QI and drew out factors affecting this. This analysis, together with the macro-level context, was written as a country report. The five country reports were then translated into English, as the working language.

Two researchers worked independently to code themes in the five country reports on requirements and strategies related to hospital finance and quality.^19,22^ Iterative testing of themes and discussion between the researchers led to cross-checking and allowed for inclusion of new insights. From this, we compiled a description and classification of the strategies exhibited by the hospitals in the sample, structured according to the main dimensions identified by Oliver.^4^

### Validity and reliability of data collection

Regular meetings of the research teams from each of the partner countries ensured that the fieldwork was conducted in the different countries consistently and reliably. Ongoing discussions amongst researchers and an advisory board provided opportunities for reflexivity and the development of insights into the effect of context on QI.

## Results

Analysis of the national level in each country shown in Table 1 provided context for the analysis of the hospital case studies and, in particular, the basis for characterizing the strength of external demands for cost and quality (see Table 2, second column). Table 2 summarizes how hospitals responded to these competing demands, including notable hospital characteristics and our identification of hospital strategy types.

**Table 1.** Summary of national policy-level characteristics in each country related to funding and quality of health care at the time of the research in 2011.

Year: 2011	England	Netherlands	Norway	Portugal	Sweden
Population	61.3 m	16.5 m	4.8 m	10.6 m	9.3 m
Austerity measures in financial years 2010 and 2011	Budget cuts 0.2% and 2.2%	Limited to 2.5% growth	Budget cuts 0.8% and 0.2%	Budget cuts 13% and 7%	Costs limited to 9.5% GDP leading to growth of approx 1–3%
Funding (see note below)	Tax-based. Mainly publicly funded	Mix of taxation and insurance	Tax based	Tax based	Tax based
Remuneration related to quality of care	Hospitals remunerated through contracts with commissioners for volume and quality	Insurance companies different quality requirements in contracts. Hospitals manage multiple demands	Main hospital funding from government through regions not linked to quality but waiting times guarantee with financial penalties	Hospitals remunerated in block funds from government with activity targets. 4% budget incentivized for delivering national quality and efficiency targets	Financing through County Councils – volume and some quality measures/incentives. Recent schemes of payment from government in relation to access
Regulatory framework for quality	Explicit focus on quality, targets and use of financial rewards and penalties. Hospital licensing in place through the national Care Quality Commission	Explicit focus on quality, targets and use of financial rewards and penalties. Hospital accreditation is in place. Many bodies involved in QI	Regional with some oversight. Requirement to have systems in place to control quality with discretion about how to do this. No accreditation system	Regional with some oversight. Requirement to have systems in place to control quality with discretion about how to do this within boundaries. Hospital accreditation is in place	Autonomous County Councils/Regions – decision making. Guidelines developed centrally but few requirements and targets. No accreditation system
Reforms underway (2012)	Major structural changes in purchasing to devolve responsibilities to GPs	Minor reforms to payment for performance to strengthen competition, requirement for hospitals to have a safety system, insurers to use care quality in purchasing decisions	Major structural reform involving patient pathways, roles of municipalities, funding, administrative, service development	Major structural reforms to primary and ambulatory care, long-term care and hospital management and inpatient care	Major reforms to increase diversity of providers change in ownership of primary care centres and pharmacies
Public access to information about quality of care	Large amount of information available to the public	Large amount of information available to the public	Growing amount	Very little	Growing amount

GDP: gross domestic product; QI: quality improvement; GPs: general practitioner.

None of the hospitals in the study used private treatment income to supplement or take the place of publicly funded care.

**Table 2.** Summary of hospital strategies, response descriptor and characteristics.

Country and hospital and resources	Strength of external demands for cost and quality	Notable hospital characteristics	Hospital leaders response^a^	Hospital strategy type
England A 2200 beds 12,000 staff Teaching	High (both)	Unstable finances; changes in leadership	Acquiescence to financial demands and a degree of avoidance and defiance with regard to the quality demands	Short term
England B 1025 beds 7500 staff	High (both)	As above	As above	Short term with attempts at the medium term but non-aligned
Portugal A 1300 beds 1700 staff Teaching	High – cost Medium – quality	As above	As above	Short term
Portugal B 585 beds 1300 staff	High – cost Medium – quality	Stable leadership	Moved from a position of acquiescence and avoidance to one of compromise and manipulation	Medium term, aligned
Norway A 300 beds 2300 staff Nurse teaching	Medium – cost Medium – quality	Leaders distracted by other events, but stable	Acquiescence to financial demands with a degree of both avoidance and defiance for quality demands but less so than in organizations with short-term measures	Medium term, non-aligned
Norway B 1100 beds 11,000 staff Teaching	Medium – cost Medium – quality	Stable leadership	Moved from a position of acquiescence and avoidance of external demands for quality and costs to one of compromise and manipulation	Medium term, aligned
Netherlands A 710 beds 3700 staff Teaching	Low – cost High – quality	Stable finance, performance and leadership over time	Compliance and compromise with quality and cost demands, leaders having engaged in dialogue to align the different demands	Longer term (embedded)
Netherlands B 540 beds 2.600 staff Teaching	Low – cost High – quality	Leaders distracted by other events	As Norway A	Medium term, non-aligned
Sweden A 500 beds 3300 staff Teaching	Low – cost Medium – quality	Stable finance, performance and leadership over time	Compliance and compromise with quality and cost demands, leaders having engaged in dialogue to align the different demands	Longer term (embedded)
Sweden B 640 beds 4080 staff Teaching	Low – cost Medium – quality	As above	As above	Longer term (embedded)

a Based on the typology in Oliver.^4^

All hospitals had QI initiatives underway, and improving the quality of care was important to the work of hospital leaders. However, how QI was managed and supported, and which initiatives or programmes were prioritized, differed between hospitals. Analysis identified four predominant strategies for meeting external demands for both financial balance and QI, described below. We found hospitals attempting to move between these strategy types, and we found hospitals applying more than one strategy at the same time. However, we consider the strategy that predominated in each organization.

### Short-term (immediate) cost-saving measures and their impact on QI

Short-term measures were found in both hospitals in England and in one in Portugal. In these organizations, applying the responses described by Oliver,^4^ we found acquiescence to financial demands by leaders, and a degree of avoidance and defiance with regard to the quality demands.

In England, external demands for quality were multiple, the strength of the cost-cutting demands was high and leadership instability was evident. Hospital leaders focussed their efforts on cost-saving measures often at the expense of QI, making an exception to deliver the quality demands that had the potential to impact adversely on the hospital (for example, those that if not delivered would reduce the hospitals’ income from payers; or the requirements of regulators that could affect the hospitals’ future viability). Short-term measures were also evident in Portugal A, with high demands for cost reductions, but hospital finances had been under pressure for some time, and there had been changes in leadership.

In England B, staff described how the organization focused on making improvements identified by the national regulator but then suddenly lost this focus in the face of necessary financial savings: ‘We lived and breathed the [regulator] until last September, but it hasn’t, I have to be honest, I don’t think it’s continued with the same focus because finances have been a big issue and they have taken precedence …’

In Portugal A, interviewees referred to a ‘trade-off’ between QI and reducing costs. Here, hospital leaders were described as ‘calibrators’ of the tension between reducing costs and maintaining the quality of services.

These hospitals had invested less in training for QI and had fewer external links to help staff in QI work. The short-term measures involved cancelling study leave and freezing vacancies, leaving permanent staff with no ‘slack’ time to consider QI activities.

### Medium-term (two- to three-year) strategies where finance and quality goals were not aligned

In organizations with non-aligned medium-term strategies, applying the model by Oliver,^4^ we found leaders acquiescing to financial demands with a degree of both avoidance and defiance for quality demands but less so than in organizations with short-term strategies. In these organizations, managerial attention was diverted from QI by intra-organizational dynamics.

This response was found in Netherlands B and in Norway A (this hospital had been operating a longer term QI strategy, but this was disrupted by short-term problems). Where external demands for QI were multiple and where senior leaders appeared unable to prioritize or refuse certain demands, the results were target overload and staff becoming frustrated and overwhelmed with monitoring and measuring multiple tasks. For example, Netherlands B was described as trying to do everything at once, resulting in too many QI activities and no overview, with one interviewee saying: ‘ … nothing is done properly anymore, and there is not enough time to evaluate the activities/actions one is supposed to control’.

Where the reorganization of services was not clearly linked to QI, but there was an obvious financial benefit, this resulted in local opposition. For example, the restructuring of clinical services in Norway A encountered local service user opposition which was described as drawing hospital leaders into ‘an ongoing hospital battle’.

### Medium-term (two- to three-year) cost-saving measures where financial and quality goals were aligned

By contrast, in hospitals where leaders had begun to link reducing costs with improving quality through process redesign to improve efficiency, reduce waste and stream-line care, the changes were positively associated by staff with improvements in quality. Using the model by Oliver,^4^ these organizations appeared to have moved from a position of acquiescence and avoidance of external demands for better quality and lower costs to one of compromise and manipulation. That is, they were actively working to influence (in a positive sense) their external institutions and the demands placed on them for cost and quality.

The organizations (Norway B, Portugal B) had managed to gain support from internal and external stakeholders for service changes aimed at improving quality and reducing costs. The challenge of ensuring all stakeholders who were involved was recognized by the President of the Board in Portugal B:

Our main concern now is how we can improve sustainability without losses in the system, in processes and in outcomes in terms of quality … we realise that it will probably entail the restructuring of the hospital, but above all will involve major involvement and participation by all.

Staff in Norway B described different conversations about QI and finance taking place over time, from when the organization was in financial difficulty to when the organization had achieved financial balance and quality could then be considered: ‘My clinic went through major changes in 2007-2008, and we focused on managing by targets, … currently we talk about economy, yes, … but not economical aspects only. We talk a lot about professional development, patient quality, patient safety …’

### Strategies developed over the longer term (three to five years and more) relating costs and QI

The response of hospitals (Oliver^4^) with longer term strategies was one of compromise – considering how to embed the requirements into the work of the hospital. These hospitals were characterized by stability in leadership, finance and operational performance over time.

Three hospitals had been working over many years to embed QI in the culture of the organization (Sweden A and B, Netherlands A). These were in countries where there was less pressure to reduce costs from the national level but where there were cost pressures locally. These organizations had been able to invest in training and developing staff in QI work, and quality was seen as part of the everyday work of all staff. As one interviewee in Sweden B said: ‘The hospital has no exact figures for QI work since it is considered to be part of everyone’s responsibility’.

In Swedish hospitals, the quality and cost requirements were broadly aligned at the county level. Here, senior leaders had engaged in dialogue with the external organizations to influence and align their different demands (manipulation^4^). In Netherlands A, aligning external demands was undertaken by senior hospital leaders.

## Discussion

Considering the model by Oliver,^4^ hospital responses to financial demands were more likely to be acquiescent where the demands were strong and where the hospital was already in financial difficulty. In these hospitals, as in those with non-aligned cost and quality strategies, there was a degree of ‘avoidance’ and ‘defiance’ with regard to external demands for QI. Here, leaders focussed on delivering the quality demands that affected the reputation or the funding of the hospital. As hospitals moved towards strategies that were medium and long term and where leaders were able to align cost and quality requirements into an overall QI strategy, the response to both cost and quality demands became one of compromise and manipulation (meaning positively influencing the external demands).

### The role of local hospital characteristics in filtering external demands

Greenwood and Hinings^13^ found that not all organizations experience conflicting institutional demands in the same way. They describe how external demands are filtered and enacted differently by different organizations. This was found in our study where the hospitals in the same country (Portugal, Norway, the Netherlands) each displayed different strategies, despite being in the same health care system with similar external demands. The different hospital responses were related more to local factors, for example, whether or not the hospital had experienced financial difficulties over time (Portuguese hospitals were the strongest examples of this) and whether or not there was sufficient managerial ability to negotiate and align internal and external demands into a coherent QI strategy that staff could support (Dutch hospitals were the most obvious example here).

Hospital responses to the difficult challenge of managing competing external demands to reduce costs and improve quality often appeared as ‘messy’ and/or ‘emergent’ in the short to medium term. In these hospitals, for example, England A and B, there had been frequent changes in leadership, and the top team was not ‘settled’. However, the hospitals with longer term integrated QI strategies were working in an environment where both the finances and the leadership of the hospital had been stable for many years. This supports the arguments by Delmas and Toffel^23^ and Pache and Santos^14^ that knowledge of local characteristics is vital in understanding how and why the different responses proposed by Oliver^4^ are found in different institutions.

Echoing other studies,^24^ our study showed that organizations undergoing periods of uncertainty such as downsizing often lost their focus on QI. Uncertainty within an organization has been highlighted as an important factor having a negative influence on the course and success of change programmes.^25^ The findings are also in line with the concept of a ‘receptive context’ for change, often referred to as ‘organizational readiness for change’.^26,27^ Studies investigating organizational change in health care^27^ suggest that a better appreciation of these local factors is likely to increase the chance of change succeeding.^28^

### Progression and movement between strategies

For most hospitals in the study, staff perceived that quality would slip off the agenda as financial restrictions were applied. This indicates that hospitals can move down as well as up the ladder of strategies, changing their response to external pressures from compromise and compliance to acquiescence and defiance as circumstances change. This fragility has been found in other studies, in which the so-called crumbling edge of quality sees attention to long-term quality issues fall away rapidly as short-term financial exigencies assume priority.^29^

Questions arise as to how or whether it is possible to prevent this happening. The question also arises as to what conditions would enable a progression from short-term measures for cost and quality to a long-term integrated QI strategy. In hospitals taking short-term measures, there was almost a vicious circle at play whereby time and effort for QI was being cut and leaders focused only on the immediate quality requirements of regulators. By contrast, hospitals with a long-term strategy had well-trained staff with time for QI and a well-developed understanding of the relationship between cost and quality across the organization. Moving out of short-term measures clearly needs more than just a resolution of the financial position; it also needs investment in building QI skills and in developing and embedding the linkages in the organization between cost and quality.

## Limitations

National-level and local fieldwork data were collected and analysed by researchers in their own language, then written into a country report which was translated into English. These reports were used in the analysis so some data may have been ‘lost in translation’ despite a common framework for collecting and analysing the data and rigorous checks.

Case studies are a useful method to apply in developing organizational theories.^17,30^ However, since we studied only two hospitals in each of the five countries, the study is unlikely to include the complete range of possible configurations of conditions. For example, it is known that in England there are hospitals that have aligned cost and quality demands into coherent long-term strategies whilst working with the same external demands. Rather, the study provides unique, detailed cross-country hospital-level data, to explore the utility and implications of institutional theory and to understand how different health care organizations manage external pressures on quality and costs.

## Conclusions

Drawing on institutional theory, our findings indicate that how hospitals respond to financial and quality challenges is dependent upon three factors: first, the coherence of the demands from external institutions; second, managerial competence to align demands and last but not least, a settled leadership team that ‘stays the course’. Those with all three in place are more likely to respond to external demands with compromise (considering how to move the organization towards the demands over the longer term) and manipulation (working to influence external demands in a positive way to improve quality). Where these factors are not in place, leaders are more likely to respond with acquiescence (habitual conformance) and/or defiance and avoidance (symbolic or rhetorical conformance that is ‘decoupled’ from actual operations).

What are the lessons for policy makers? The typology provides a basis for policy makers to consider how hospitals may respond to policy challenges. Where there is potential at the policy level to manipulate or shape external demands in order to balance/integrate quality and cost demands, this can only help hospitals to deliver QI. However, in countries where there are multiple demands from multiple players, it rests on the skills of hospital leaders to bring cost and quality demands together and align them into a coherent QI strategy. Here, the task at the policy level is to ensure hospital leaders are well trained and supported in this complex task.

What are the lessons for hospital leaders? The typology provides a basis for hospital leaders to reflect on their response to external pressures. The development of leaders’ skills in translating external requirements into implementation plans with internal support in each hospital is clearly vital. Given the importance of local factors, clearly hospitals cannot simply copy success from elsewhere. A longer term plan is required that works to increase QI skills, develop a culture where QI becomes embedded, and which links cost reduction measures to streamlining and improving care. Importantly, leaders need to be skilled in negotiation to enable them to work with external organizations to shape the demands.

This study has identified the need for more knowledge about the managerial abilities and skills needed to balance trade-offs and manage the interface between the national and local levels whilst also improving quality. An examination of the ‘work’ of local health care leaders in QI using a wider body of institutional theory would be helpful. A longer longitudinal study would also be valuable to study hospitals as they attempt to move from the short-term to longer term strategies.
